# A framework for assessing the risk of resistance for antimalarials in development

**DOI:** 10.1186/1475-2875-11-S1-P23

**Published:** 2012-10-15

**Authors:** Xavier C Ding, David Ubben, Timothy NC Wells

**Affiliations:** 1 Medicines for Malaria Venture, 20 rtede Pré Bois, CH 1215 Geneva, Switzerland

## 

Because they kill sensitive organisms, anti-infective agents are bound to exert an evolutionary pressure toward the emergence and spread of resistance mechanisms. Common to all infectious diseases, this vicious circle is especially acute for malaria. *P. falciparum* resistance to chloroquine and sulfadoxine-pyrimethamine became so widespread that these former first-line treatments had to be abandoned [1]. Today, in certain areas, *P. falciparum* parasites appear to gradually lose their sensitivity to artemisinin derivatives, on which are based the current therapies for both uncomplicated and severe *P. falciparum* malaria [2,3]. New classes of antimalarial medicine are urgently needed to stay ahead in the resistance arms race. These should be designed not only to overcome existing resistance mechanisms, but also to prevent the emergence of *de novo* resistance for as along as possible.

Cell-based screening methods have led to a renaissance of new classes of anti-malarial compounds [4], offering us the potential to select and modify molecules based on their resistance potential. In order to quantitatively assess this potential in *P. falciparum*, we developed a standardized *ex vivo* methodology that can be applied during the early phases of the drug development process. Cross-resistance is evaluated through a panel of specific multi-drug resistant strains designed to cover all genetically validated resistance mechanisms known to occur in the field. Second, the genetic ability of *P. falciparum* to evolve a genetically encoded resistance mechanism is quantified by measuring the minimal inoculum for resistance (MIR), that is the minimal number of parasite from which a resistant mutant is likely to be selected *ex vivo* by a constant low level of drug pressure. Further, the generation of resistant parasites possibly facilitates the understanding of the compound mode-of-action and permits the identification of resistance markers, which are essential for resistance monitoring during the clinical development and post-marketing surveillance phases.

Altogether, these and other parameters, such as resistant parasite fitness and gametocyte production, define a comprehensive profile, which allows the identification of overt risks and the active prioritization of the most robust antimalarials in a cost-effective manner.
